# In-Vitro Evaluation of Antioxidant, Antiproliferative and Photo-Protective Activities of Benzimidazolehydrazone Derivatives

**DOI:** 10.3390/ph13040068

**Published:** 2020-04-15

**Authors:** Anna Baldisserotto, Monica Demurtas, Ilaria Lampronti, Massimo Tacchini, Davide Moi, Gianfranco Balboni, Silvia Vertuani, Stefano Manfredini, Valentina Onnis

**Affiliations:** 1 Department of Life Sciences and Biotechnology, University of Ferrara, Via Fossato di Mortara 17-19, I-44121 Ferrara, Italy; anna.baldisserotto@unife.it (A.B.); mv9@unife.it (S.M.); silvia.vertuani@unife.it (S.V.); 2 Department of Life and Environmental Sciences, Unit of Pharmaceutical, Pharmacological and Nutraceutical Sciences, University of Cagliari, University Campus, S.P. n° 8, Km 0.700, I-09042 Monserrato (CA), Italy; monicademurtas@tiscali.it (M.D.); davide.moi2@gmail.com (D.M.); gbalboni@unica.it (G.B.); 3 Department of Life Sciences and Biotechnology, Section of Biochemistry and Molecular Biology, University of Ferrara, Via Fossato di Mortara 74, I-44121 Ferrara, Italy; ilaria.lampronti@unife.it; 4 Department of Life Sciences and Biotechnology, Section of Pharmaceutical Biology, University of Ferrara, Piazzale Luciano Chiappini 3, I-44123 Malborghetto di Boara (FE), Italy; massimo.tacchini@unife.it

**Keywords:** benzimidazoles, hydrazones, polyhydroxylated compounds, antioxidant activity, photoprotective agents, antiproliferative activity

## Abstract

In the search of multifunctional compounds we designed benzimidazole derivatives endowed with phenolic hydroxy groups and a hydrazone moiety as potential radical-scavenger and the antioxidant agents. The target molecules have been prepared by a simple synthetic procedure and tested for their antioxidant activity by DPPH, FRAP, and ORAC test, for photoprotective activity against UV rays and for antiproliferative activity against Colo-38 melanoma cells. Furthermore, two different dermocosmetic formulations were prepared with the compounds endowed with the best antioxidant and photoprotective profile and their release from formulation evaluated using Franz Cells system. High antioxidant activity is related to the presence of at least two hydroxy groups on arylidene moiety of benzimidazoles. Structure activity analysis revealed that the position of hydroxy groups is crucial for antioxidant activity as well as the presence of a 2-hydroxy-4-(diethylamino)arylidene group. The same correlation pattern was found to be related to photoprotective activity resulting in an UVA Protection Factor better than the commercial solar filter PBSA and antiproliferative activity against melanoma cells without producing cytotoxicity on normal keratinocytes. The release analysis indicated that high antioxidant activities are achieved with limited release at concentration compatible with the use as UV sunscreen filter.

## 1. Introduction

The heterocyclic benzimidazole nucleus is widespread in nature and present in several bioactive compounds. Benzimidazole is also a privileged ring in medicinal chemistry and this pharmacophore is included as a key part in antimicrobial, anticancer, acetylcholinesterase, antiprotozoal, anti-inflammatory, analgesic, antihistaminic, antiallergic, enzyme inhibitory, antimalarial, antitubercular, and antiviral agents [1,2,3,4,5,6,7]. Several benzimidazole-based compounds have been also reported to possess antioxidant activity [1,8,9,10,11,12]. Reactive oxygen species (ROS) and reactive nitrogen species (NOS) are well known as both harmful and beneficial species [13]; however, when unbalance occurs between endogenous antioxidant defense and ROS oxidative stress conditions occurs. This latter has been related to an extensive range of diseases including cardiovascular, inflammatory, neurodegenerative, and autoimmune ones [14,15]. Overproduction of reactive oxygen species can be responsible for damage to vital cell components, especially to DNA, lipids, and proteins. It is known that the use of antioxidants is beneficial in the prevention or delay of numerous diseases associated with oxidative stress including cancer, Alzheimer’s and other neurodegenerative diseases, as well as atherosclerosis [16,17,18,19]. It is also known that antioxidants such as phenols break down oxidation chains by reactions with peroxy radicals. Tocopherol, p-coumaric acid, ferulic acid, and caffeic acid are the best-known phenolic antioxidant agents. On the other hand, also hydrazone moieties are endowed with potent antioxidant potency [20,21,22,23]. Based on these findings and as a continuation of our works on antioxidant agents [24,25,26], here we report and discuss the antioxidant properties of benzimidazolehydrazones containing a phenolic group and their structure–activity relationships (SAR). The studied molecules have been synthesized and chemically characterized by us during a previous synthetic study [27], but not fully investigated for their activity. The designed molecules are characterized by a phenolic hydroxyl group and hydrazone moiety whose azomethine fraction plays a critical role in antioxidant activity [28] and possibly contribute to radical-scavenging activity and the antioxidant property. 

## 2. Results and Discussion

### 2.1. Chemistry

The synthetic approach to hydrazones **3**–**15** compounds was based on a previously described two steps procedure [27] that gave high yields starting from commercially available ethyl 1H-benzo[d]imidazole-2-carboxylate (**1**) that was converted in the corresponding hydrazide **2** which was coupled with the appropriate hydroxyarylaldehydes in ethanol to afford benzimidazolehydrazones **3**–**15** (Table 1).

### 2.2. Antioxidant Activity

The evaluation of the antioxidant properties of the benzimidazole hydrazones **3**–**15** was achieved by 1,1-diphenyl-2-picrylhydrazyl radical scavenging activity (DPPH), ferric reducing antioxidant power (FRAP), and oxygen radical absorbance capacity (ORAC) methods. Results, shown in Table 1, are expressed as μmolTE/g for DPPH, FRAP, and ORAC tests and are compared to the reference compound Trolox, also used as a standard, and ferulic acid was used as positive control. For the best interpretation of the results of the DPPH test, each compound was tested at the concentration capable of inhibiting 50% of the radical, except for compounds **3**, **4**, and **14** which data refer to the limit of quantification.

The radical scavenging activity of the benzimidazole hydrazones **3**–**15** that emerged from DPPH results indicates that the hydrazones bearing a single hydroxy group on the aryl ring (compounds **3**–**5**) showed weak antioxidant activity. By introducing a second hydroxy group in the 4-position (hydrazone **6**) the antioxidant capacity increase of about 8-fold as compared to 2-hydroxyphenyl analog **3**. The shift of 4-hydroxy group into 5-position to give compound **7** led to further increase in activity, about 400-fold as compared to hydrazone **3**. The introduction of a third hydroxy group at 3-position of hydrazone **6** to give compound **8** produced about 55-fold enhancement in activity. On the contrary the introduction of a third hydroxy group at 6-position (compound **9**) did not modify the antioxidant potency that is practically unchanged as compared to hydrazone **6**. Taken together these results indicate that high antioxidant activity is related to the position of the hydroxy groups rather than to their number.

The introduction of alkoxy groups (hydrazones **10** and **12**) at 4-position of compounds **3** and **4** produced about 10-fold increase in activity. However, the presence of alkoxy group at 3-position (compound **11**) led to slight reduction in activity as compared to hydrazone **3**. The replacement of the 5-hydroxy group of compound **7** with halogen atoms as in hydrazones **14** and **15** led deep reduction in activity with DPPH values similar or lower than hydrazone **3**. The replacement of 4-hydroxy group of hydrazone **6** with a dimethylamino moiety (hydrazone **13**) produced about 5-fold increase in activity.

FRAP analysis, according to the results obtained from the DPPH test indicated that high antioxidant activity is related to the position of the hydroxy groups rather than to their number. In fact, the 2-hydroxyphenyl hydrazone **3** confirmed its poor antioxidant activity. Nevertheless, the shift of the hydroxy group into the 3- or 4-position (hydrazones **4** and **5**) led to about 20-fold increase in activity. By introducing a second hydroxy group at the 4-position (hydrazone **6**) the antioxidant capacity increase of about 6-fold as compared to 2-hydroxyphenyl analog **3**. The shift of 4-hydroxy group into 5-position to give compound **7** led to further increase in activity, about 100-fold as compared to hydrazone **3**. The introduction of a third hydroxy group at 3-position or 6-position of hydrazone **6** to give compounds **8** and **9** produced about 35-fold and 14-fold enhancement in activity respectively. The introduction of alkoxy groups on the phenol ring of compounds **3** and **4** (hydrazones **10**–**12**) produced a moderate increase in activity.

The compounds that showed the most interesting profile following the DPPH and FRAP tests were also subjected to a further test (ORAC) to outline a complete antioxidant activity profile.

In the ORAC assay the 4-hydroxyphenyl derivative **5** demonstrated the best antioxidant capacity towards the peroxyl radicals (ROO^-^). The shift of hydroxy group into 3-position (hydrazone **4**) produced slight reduction in activity. Contrary to DPPH and FRAP assays in ORAC test the dihydroxyphenyl and trihydroxyphenyl derivatives showed lower activity as compared to the monohydroxyphenyl analogs. Furthermore the 2-hydroxy-4-(diethyl)aminophenyl derivative **13** did not confirmed the high antioxidant capacity showed in DPPH and FRAP.

### 2.3. Evaluation of UV-Filtering Parameters

On the hydrazones **4**–**9**, **12**, and **13** showing the best antioxidant profile, in vitro tests were conducted to determine the parameters fundamental for evaluation of the filtering power: SPF value (Sun Protection Factor), UVAPF0 (UVA Protection Factor Value), UVA/UVB ratio, and critical wavelength (λc) (Table 2). The Solar Protection Effectiveness Evaluation System specifies a SPF primarily representing a measure of UVB protection [29] is related to the UV absorption of substances.

**Figure 1:**
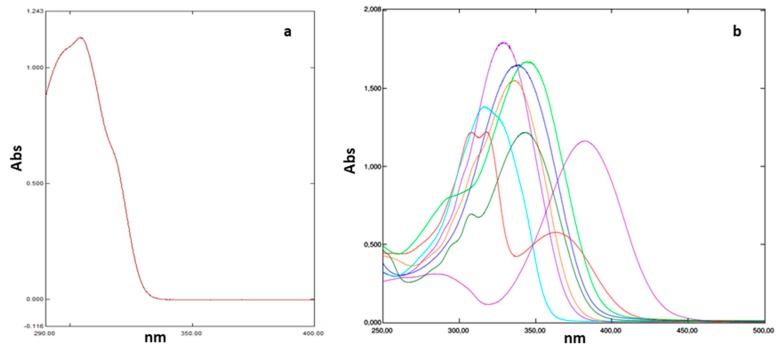
Panel (**a**), UV spectra of the reference PBSA; panel (**b**), UV absorption spectra of hydrazone derivatives: **4** (light blue), **5** (violet), **6** (dark green), **7** (red), **8** (blue), **9** (light green), **12** (orange), **13** (fuchsia).

The spectra of hydrazones **4**–**9**, **12**, and **13** were recorded in the 250 and 500 nm range (Figure 1) and compared with the commercial filter phenyl benzimidazole sulfonic acid (PBSA). The spectrum of the reference commercial filter PBSA is characterized by maximum absorption peak at 302 nm and any absorption in the UVA region. The comparison of UV spectra of the reference sunscreen filter PBSA and hydrazones endowed with the best antioxidant activity showed the λmax of the hydrazones **4**–**9**, **12**, and **13** shifted towards longer wavelengths, as compared to PBSA. Above 350 nm the absorbance is reduced almost to zero (Figure 1). Furthermore, the absorption curves of the analysed compounds have a wider range than PBSA. The bathochromic shift observed for all the analysed hydrazones, is related to the presence of auxocrome groups on the arylidene moiety. The shift of the absorption band towards longer wavelengths is particularly pronounced in the 2-hydroxy-4-(diethylamino)phenyl derivative **13**. The values related to the filtering parameters were extrapolated from the UV absorption spectra.

The hydrazones were also evaluated for their UV-B filtering capabilities by the Diffey-Robson in vitro method [30]. Table 2 shows the data obtained from the analysis of the tested hydrazones as well as reference PBSA. In vitro tests are usually performed during the preliminary phases of development of sunscreen molecules to determine the UV protection potential. Although a new method has been established and standardized in the ISO 24443: 2012 for the in vitro evaluation of UVA protection, there is no equivalent recognized in vitro method for evaluating protection in the UVB range. Among the proposed tests the in vitro method of Diffey-Robson is the most known and widely applied [30]. Table 2 shows the data obtained from the analysis of the tested hydrazones as well as reference PBSA. According to the EU recommendation of 2006 (2006/247/EC) a solar product is considered to have broad spectrum when its lambda critical (λc) value is higher than 370 nm. In this respect only hydrazones **7**, **9**, and **13** satisfied this requirement.

However, among broad-spectrum derivatives, the SPF value is noticeable only for compounds **7** and **9**. The UVA/UVB ratio, as defined by the aforementioned EU recommendation, should be at least 1/3. All hydrazones, except **4** and **7**, showed UVA/UVB ratio greater than 1 and satisfied this requirement, meaning an absorption mainly in the UVA region. The last value, determined according to ISO-24443, is UVA Protection Factor (UVAPF0). All hydrazones are endowed with UVAPF better than PBSA. The hydrazone **9** showed the best UVAPF0 value, about 10-fold than the reference PBSA. As mentioned above only hydrazones **7**, **9** and **13** had λc value higher than 370 nm. Regarding the photoprotective activity, in general it can be said that the hydrazone derivatives series showed a better in vitro SPF profile than that of the reference PBSA sunscreen filter. It has been observed that the SPF parameter does not seem to be always influenced directly in proportion to the number of hydroxyls present on the substituent, a correlation that is equally valid for the UVAPF parameter. The presence of a methoxy group or the 2-hydroxy-4-(diethylamino) portion positively influences the filtering parameters.

### 2.4. Evaluation of Release In Vitro

**Figure 2:**
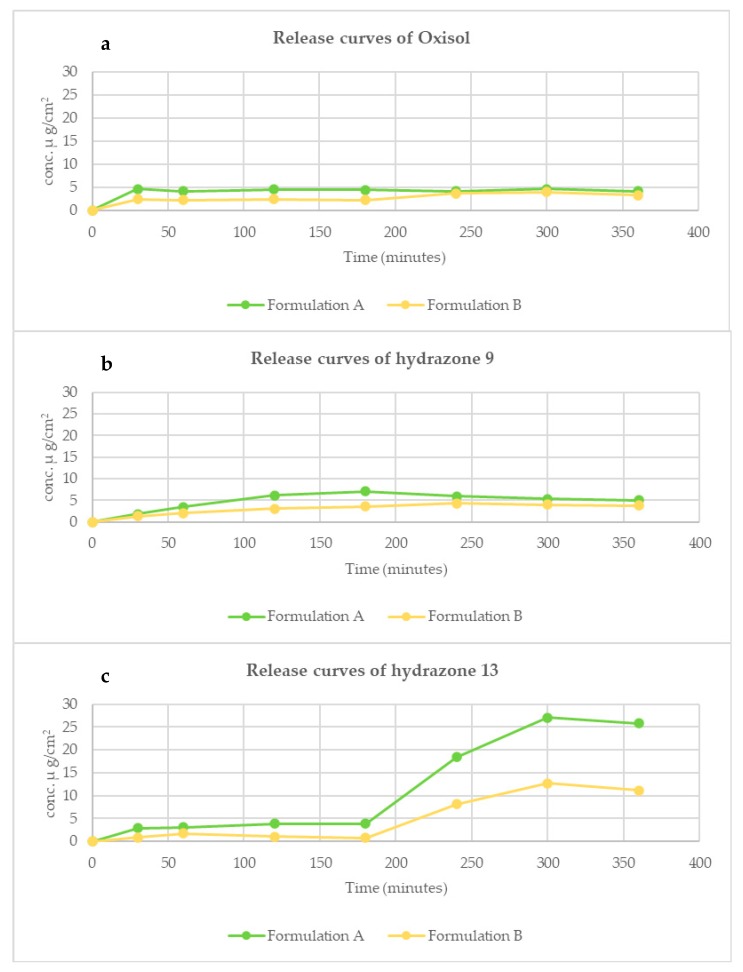
Permeation profiles of Oxisol (panel (**a**)), and hydrazones **9** (panel (**b**)) and **13** (panel (**c**)). Line green corresponds to formulation A (optimized for skin adsorption), line yellow corresponds to Formulation B (optimized to best solubilize the active in formula).

The hydrazones **9** and **13** showing the best dualistic profile were selected to evaluate their potential, as antioxidant UV filtering ingredients for dermocosmetic formulations, by in vitro experiments. To this end two topical formulations, differing in degree of polarity, have been prepared based on our previous background in the investigation on Oxisol [31]: Formulation (A), designed for the antioxidant activity and therefore for a high release of the active to the skin, and Formulation (B) designed instead for a sunscreen filter and then aimed to obtain avoid release of the active ingredient due to its high affinity for the formulation. The release study was carried out using Franz Cells, the most commonly used in vitro approach to evaluate the release of active ingredients from semi-solid preparations. The amount of hydrazones, **9** and **13**, and Oxisol released in the receiving chamber was sampled within 6 h and the resulting release curves are shown in Figure 2.

The release curves obtained Franz cell assay of hydrazones **9** and **13** compared with the reference Oxisol (Figure 2) maintain the same trend both for the reference Oxisol and for the hydrazone derivatives **9** and **13**. In the case of Oxisol and compound **9** the delta tends to shrink after 240 min. The graph of compound **13** presents trend and delta between the two curves almost constant; however, after the first 180 min the release from formulation A tends to progressively increase, until it reaches a difference of about 15%. This result, analogous to those obtained previously [25], supports the possibility of modifying the release of the active ingredient from the formulation by changing only the percentages of the components of the formula ad hoc designed, and its composition, depending on the function desired of the active component.

### 2.5. Antiproliferative Activity

Polyphenols are known for their activity against melanoma [32], a malignant and aggressive tumor associated with intense exposure to UV radiation. For this reason, once the compounds of the series with an excellent antioxidant profile associated with filtering capacity were identified, it was decided to evaluate their anti-proliferative activity on melanoma cell cultures (Colo-38). On the most active compounds the antiproliferative activity was tested also on human skin keratinocytes HaCat using a previously described method [26].

In the performed experiment, the cells were seeded at a concentration of 40,000 cells/mL and cultivated in the presence of increasing concentrations of the compounds examined (from 0.01 to 500 μM). Cell counting was executed after 48 h and 72 h from treatment. For each hydrazone the value of IC_50_ (concentration value necessary for the inhibition of 50% cell growth) was calculated starting from at least three independent experiments. The results of the preliminary assay, which aimed to identify eligible candidates as potential active drugs against melanoma, are listed in Table 3.

Among the best antioxidant hydrazones tested against human melanoma Colo-38 cells, compounds **12** and **13** emerged, which showed the best anti-proliferative effect with IC_50_ values respectively equal to 0.84 and 0.50 µM. These hydrazones were also tested on immortalized human keratinocytes HaCat cells, in order to verify a possible selectivity for tumor cell lines. Both hydrazones **12** and **13** demonstrated selectivity for cancer cells with IC_50_ values against HaCat cells (53.16 ± 4.33 and 5.03 ± 0.76 for **12** and **13** respectively) about 60-fold and 10-fold higher as compared to melanoma Colo38 human cells.

## 3. Materials and Methods

### 3.1. General Methods

The spectrophotometer used for antioxidant analysis is a Beckman Coulter™, DU^®^530, Life Science UV/VIS spectrophotometer, Single Cell Module. The instrument used to conduct ORAC analyzes is the Thermo Fluoroskan Ascent FL^®^ Microplate Fluorometer and Luminometer, linked to Ascent Software^®^ software for data control and processing. In the sample loading phase, 96-well plates with a black background were used. Spectrophotometric analyses for the detection of filter parameters were conducted with a UV–VIS spectrophotometer SHIMADZU UV-2600 240 V. The benzimidazolehydrazones **3**–**15** have been synthesized using our previously described procedure [27].

### 3.2. Antioxidant Activity Evaluation

#### 3.2.1. DPPH Test

The benzimidazole hydrazones 3–15 were tested against DPPH• radical following the Wang et al. method [33] modified as previously reported [24]. The 1,1-diphenyl-2-picrylhydrazyl (DPPH) radical-scavenging assay measures the hydrogen donation ability of an antioxidant to convert the stable DPPH free radical into 1,1-diphenyl-2-picrylhydrazyl. This can be evaluated by measuring the percent decrease in absorbance of the solution at 517 nm, through the color change from deep-violet to light-yellow, after the radical reaction with products to be tested. The radical-scavenging activity is expressed as inhibition ratio of initial concentration of DPPH radical and is calculated according to the formula:

Inhibition percentage (Ip) = [(AB − As)/AB] × 100(1)

where AB and As are, respectively, the absorbance values of blank reaction and of the tested sample. The DPPH solution is prepared by dissolving 4 mg of DPPH in 100 ml of MeOH and then the solution is stirred in the dark for 30 min. The standard solutions of Trolox are prepared in MeOH in a range between 4–95 nmoles, while sample solutions starting from 0.2 mg/mL to 0.004 mg/mL. After 1.5 mL of the DPPH solution has been added to 0.750 mL of the sample solutions proper diluted/standard/control, it is reacted for 30 min in the dark. Samples absorbance measurements were evaluated with a UV-VIS spectrophotometer at fixed wavelength of 517 nm. Blank sample was prepared adding methanol to DPPH solution and Trolox (6-hydroxy-2,5,7,8-tetramethylchroman-2-carboxylic acid) was used as standard reference to achieve a calibration curve. The results are expressed as μmol TE/g corresponding to an inhibition of the radical equal to 50%, except for some compounds.

#### 3.2.2. FRAP Test

The ferric ion reducing ability of hydrazones **3–15** was measured reading the absorbance of the reaction mixture at 593 nm, according to the method described previously [34]. The analysis reagent was freshly prepared by mixing the subsequent solutions in the fixed ratio 10:1:1 (v/v/v): 1) 0.1 M Acetate buffer, pH 3.6; 2) TPTZ (2,4,6-tripyridylstriazine), 10 mM in 40 mM HCl, 3) FeCl_3_, 20 mM. To a 1.9 mL of reagent were added 0.1 mL of sample proper diluted or solvent when blank was performed. Readings were done after 10 min, using a UV-VIS spectrophotometer at fixed wavelength of the absorption maximum (593 nm). It was evaluated the absorbance increase of sample solution against the absorbance of blank reaction as parameter to calculate the antioxidant activity. The antioxidant activity is given as Trolox equivalents (μmol TE/g compound) since this standard was used to perform the calibration curves.

#### 3.2.3. Oxygen Radical Absorbance Capacity (ORAC)

The scavenging activity test against the peroxyl radical was carried out based on a previously reported and modified protocol [35]. Sample solutions (mg/mL) and Trolox dilutions (40–240 μM) were prepared using Phosphate buffer solution (pH 7.4). In a 96-well black microplate (VWR) 25 μL of sample solution, Trolox dilution or phosphate buffer solution (pH 7.4) used as blank was placed in wells. Measurements of fluorescence were carried out at 37 °C and recorded at 5 min intervals up 30 min after the addition of AAPH. The ORAC values, expressed as Trolox equivalents (μmol TE/g compound), were calculated according to the method of Cao et al. [36]. The antioxidant capacity of the tested compound was quantified by integration and calculation of the area under the curve (AUC), relating it to that produced by reference Trolox.

### 3.3. Evaluation of Filtering Parameters

The method followed to determine the value of SPF in vitro is an adaptation of the official method [37]. The hydrazone solutions tested at a concentration of 0.000034 (± 0.0000033) M were prepared in methanol and the absorption spectra were recorded. The equation below was used to transform the absorbance values obtained into transmittance and calculate the SPF values in vitro:

A(λ) = Log[T(λ)](2)

SPF, UVA/UVB, UVAPF, and λ critical values were obtained elaborating the transmittance spectrum with the SPF calculator software (version 2.1, Shimadzu, Milan, Italy).

### 3.4. Preparation of Formulations

Two different standard oil/water emulsions (Formulation A and Formulation B) were prepared for Oxisol and hydrazones **9** and **13**. The two phases were brought to 60–70 ° C after adding the individual ingredients. The oil phase was then added under mechanical stirring to the aqueous phase. The selected active ingredients were added, always under constant stirring, in the cooling phase to avoid their thermal degradation. Once they reached 25 °C, the emulsions were stored in the refrigerator until analysis. The formulation compositions are the following:

Formulation A: Aqua, Ethylhexyl Stearate, Tribehenin PEG-20 Esters, Butyrospermum Parkii, Olea Europaea Oil Unsaponifiables, Oxisol or hydrazone **9** or **13**, Xanthan Gum, Caprylic/Capric Triglyceride, Cetearyl Alcohol, Dicaprylyl Carbonate.

Formulation B: Aqua, Cetearyl Alcohol, Tribehenin PEG-20 Esters, Butyrospermum Parkii, Olea Europaea Oil Unsaponifiables, Oxisol or hydrazone **9** or **13**, Xanthan Gum, Caprylic/Capric Triglyceride.

### 3.5. Franz Cells Apparatus

The protocol used for the release experiments followed the one previously reported [25] with the variation of the receiving solution: in this case in fact the receiving solution was Buffer phosphate 1M pH 11.2 for Oxisol, and pH 11.7 for compounds **9** and **13**. 0.5 g of each formulation was placed in the donor compartment in contact with the membrane positioned between the two compartments. Parafilm was used for donor compartment to avoid loss of formulation components. Experiments were conducted in a thermostated bath at 37 ± 1 ° C and under continuous stirring. The samples taken at any time, 30 min, 1 h and then every hour thereafter up to 6 h (and replaced with fresh receiving medium), were analyzed by UV-VIS spectrophotometer. For each compound was built a calibration curve at λmax.

### 3.6. Growth Inhibition Assays

Cell growth inhibition assays were carried out using melanoma Colo38 human cancer cell, [24,38,39] and skin keratinocytes HaCat [26]. Cell lines were maintained in RPMI 1640, supplemented with 10% fetal bovine serum (FBS), penicillin (100 Units mL^−1^), streptomycin (100 μg mL^−1^) and glutamine (2 mM); the pH of the medium was 7.2 and the incubation was performed at 37 °C in a 5% CO_2_ atmosphere. Tested hydrazones were dissolved in MeOH/DMSO 10% to obtain 20 mM stock solutions and diluted before cell treatment in MeOH 100%. The tested hydrazones were added at serial dilutions to the cell cultures (from 0.01 to 500 μM) and incubated for 3 days. Cells were then harvested, suspended in physiological solution and counted with a Z2 Coulter Counter (Coulter Electronics, Hialeah, FL, USA). The cell number/ml was determined as IC_50_ after 3 days of culture, when untreated cells are in log phase of cell growth. Untreated cells were placed in every plate as negative control.

## 4. Conclusions

Research aimed at the development of multifunctional molecules has been at the center of our work for years [24,25,27] and has led us to investigate different scaffolds such as benzimidazole, benzofurane and indole nuclei, which are however connected by an hydrazone linker and an aromatic nucleus, differently substituted, considering the importance of fractions suitable to extend the conjugation to the aromatic ring. All the derivatives have been tested for their potential antioxidant, UV filtering and anti-proliferative activity (in particular against melanoma cells).

The data of this latest study are reflected in the SAR data of previous works and contribute to implementing the experimental evidence found so far to obtain a multifunctional activity.

The study of benzofuran derivatives [24] revealed a correlation between the number and position of hydroxyl groups on the arylidene portion and good antioxidant capacity. A high activity had instead been found for the 2-hydroxy-4-(diethylamino)benzylidene derivative. The SAR study on indole derivatives [25] had led to the same conclusions, also underlining the contribution of a methoxy group in position 4 to the activity under consideration. The antioxidant data deriving from the study of benzimidazolehydrazone derivatives again confirm what reported by the previous series. As regards the antioxidant activity, in fact, the best profile was shown by the derivatives **7**, **8**, **9**, and **12** characterized by the presence of at least 2 hydroxy groups (7, 8, 9) or of a 2-hydroxy and a methoxy group in position 4 (12). The 2-hydroxy-4-(diethylamino) substituent, unlike the other series, does not seem to contribute to improving the activity against the peroxyl radical (ORAC test).

As regards the photoprotective capacity, in general it can be said that all the hydrazone derivatives series showed a better in vitro SPF profile as compared to the commercial reference PBSA filter. In particular, it has been observed that the SPF parameter does not seem to be always influenced directly in proportion to the number of hydroxyls present on the substituent, a correlation that is equally valid for the UVAPF parameter. The presence of a methoxy or a 2-hydroxy-4-(diethylamino) group positively influences the filtering parameters.

Cytotoxic activity against Colo-38 cells of human melanoma has led to the identification of derivatives belonging to the three different series, with a promising antitumor profile. The best IC_50_ values were obtained for the derivatives bearing the 2-hydroxy-4-(diethylamino) moiety: the IC_50_ data of the derivative **13** (0.50 µM) is in line with the values obtained from the derivatives of the previous series [24,25].

In conclusion we can affirm that the SAR study derived from the synthesis and analysis of heterocyclic structures that share the presence of a hydrazone spacer bearing an aromatic functionality variously substituted led us to identify a common profile to the different series. Specifically, this study has shown that the presence of the 2-hydroxy-4-diethylamino portion is related to the antioxidant, photoprotective and antiproliferative activity in the three series of hydrazones, and can therefore be considered the focus of the multifunctional profile of these derivatives.

## Figures and Tables

**Table 1:** Antioxidant activity of N^1^-(4-arylidene)-1*H*-benzo[*d*]imidazole-2-carbohydrazides **3–15.**

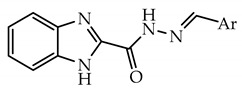				
Compound	Aryl	DPPH ^a^ µmolTE/g	FRAP ^a^ µmolTE/g	ORAC ^a^ µmolTE/g
**Ferulic acid**		4379.0 ± 9.8	6872.6 ± 9.7	15,906.4 ± 14.2
**3**	2-OH-phenyl	<23.2 ^b^	48.0 ± 2.6	-
**4**	3-OH-phenyl	<13.0 ^b^	1023.3 ± 9.4	21,808.0 ± 99.1
**5**	4-OH-phenyl	29.6 ± 0.1	1040.1 ± 11.2	30,911.3± 36.4
**6**	2,4-(OH)_2_-phenyl	200.6 ± 1.5	286.7 ± 10.3	9296.8 ± 72.3
**7**	2,5-(OH)_2_-phenyl	9387.9 ± 13.8	5330.6 ± 29.6	17,856.9 ± 35.1
**8**	2,3,4-(OH)_3_-phenyl	10,945.2 ± 38.5	10,064.6 ± 24.6	4221.5 ± 10.5
**9**	2,4,6-(OH)_3_-phenyl	192.6 ± 3.5	4071.7 ± 10.0	4098.6 ± 39.1
**10**	2-OH-4-OMe-phenyl	201.2 ± 4.9	231.9 ± 5.5	-
**11**	2-OH-3-OEt-phenyl	38.1 ± 1.6	104.5 ± 2.1	-
**12**	3-OH-4-OMe-phenyl	171.5 ± 6.1	3418.8 ± 15.2	17,170.7 ± 18.4
**13**	2-OH-4-N(Et)_2_-phenyl	1065.0 ± 5.9	3525.4 ± 13.4	754.4 ± 19.0
**14**	2-OH-5-Cl-phenyl	<< 13.29 ^b^	54.8 ± 1.3	-
**15**	2-OH-5-Br-phenyl	28.8 ± 1.2	81.7 ±2.7	-

^a^ Each value was obtained from three experiments (mean ± SE);—not tested. ^b^ LOQ limit of quantification;—not tested.

**Table 2:** In solution UV-filtering activity of selected hydrazones.

Compound	SPF	UVA/UVB	UVAPF0	λc (nm)
**PBSA**	3.42	0.29	1.04	322
**4**	11.54	0.27	1.60	343
**5**	12.32	0.75	2.95	353
**6**	4.81	1.26	4.84	366
**7**	11.23	0.32	3.24	377
**8**	8.12	2.23	6.79	366
**9**	8.34	2.27	10.65	370
**12**	8.95	1.13	3.60	358
**13**	1.57	1.18	3.99	394

**Table 3:** Effects of selected hydrazone derivatives on the proliferation of melanoma Colo-38 and keratinocyte Ha-Cat human cell lines.

Compound	IC_50_ (μM)	Compound	IC_50_ (μM)	
	Colo-38		Colo-38	Ha-Cat
**4**	362.63 ± 15.05	**8**	46.6 2 ± 3.57	
**5**	216.16 ± 40.21	**9**	459.59 ± 103.87	
**6**	46.62 ± 0.82	**12**	0.84 ± 0.03	53.16 ± 4.33
**7**	35.49 ± 0.42	**13**	0.50 ± 0.12	5.03 ± 0.76
